# Participatory AI for inclusive crop improvement

**DOI:** 10.1016/j.agsy.2024.104054

**Published:** 2024-10

**Authors:** Violet Lasdun, Davíd Güereña, Berta Ortiz-Crespo, Stephen Mutuvi, Michael Selvaraj, Teshale Assefa

**Affiliations:** a London School of Economics, Houghton St., London WC2A 2AE, United Kingdom; b Alliance of Bioversity and the International Center for Tropical Agriculture (CIAT), TARI - Selian, Dodoma Road, Arusha, Tanzania; c Alliance of Bioversity and the International Center for Tropical Agriculture (CIAT), Palmira Campus, Km 17 vía Cali, Palmira, Colombia

**Keywords:** Image-based phenotyping, Participatory plant breeding, Computer vision, On-farm variety evaluation, AI-assisted data-collection, Human centered design

## Abstract

**CONTEXT:**

Crop breeding in the Global South faces a ‘phenotyping bottleneck’ due to reliance on manual visual phenotyping, which is both error-prone and challenging to scale across multiple environments, inhibiting selection of germplasm adapted to farmer production environments. This limitation impedes rapid varietal turnover, crucial for maintaining high yields and food security under climate change. Low adoption of improved varieties results from a top-down system in which farmers have been more passive recipients than active participants in varietal development.

**OBJECTIVE:**

A new suite of research at the Alliance of Bioversity and CIAT seeks to democratize crop breeding by leveraging mobile phenotyping technologies for high-quality, decentralized data collection. This approach aims to resolve the inherent limitations and inconsistencies in traditional visual phenotyping methods, allowing for more accurate and efficient crop assessment. In parallel, the research seeks to harness multimodal data on farmer preferences to better tailor variety development to meet specific production and consumption goals.

**METHODS:**

Novel mobile phenotyping tools were developed and field-tested on breeder stations in Colombia and Tanzania, and data from these trials were analyzed for quality and accuracy, and compared with traditional manual estimates and absolute ground truth data. Concurrently, Human-Centered Design (HCD) methods were applied to ensure the technology suits its context of use, and serves the nuanced requirements of breeders.

**RESULTS AND CONCLUSIONS:**

Computer vison (CV)-enabled mobile phenotyping achieved a significant reduction in scoring variation, attaining imagery-modeled trait accuracies with Pearson Correlation values between 0.88 and 0.95 with ground truth data, and reduced labor requirements with the ability to fully phenotype a breeder's plot (4 m × 3 m) in under a minute. With this technology, high-quality quantitative phenotyping data can be collected by anyone with a smartphone, expanding the potential to measure crop performance in decentralized on-farm environments and improving accuracy and speed of crop improvement on breeder stations.

**SIGNIFICANCE:**

Inclusive innovations in mobile phenotyping technologies and AI-supported data collection enable rapid, accurate trait assessment and actively involve farmers in variety selection, aligning breeding programs with local needs and preferences. These advancements offer a timely solution for accelerating varietal turnover to mitigate climate change impacts, while ensuring developed varieties are both high-performing and culturally relevant.

## Highlights

• Leverages advances in AI image recognition to mobile-phone based digital phenotyping tools for breeding programs in the Global South
• Achieves improved trait accuracy and objectivity on breeder plots relative to manual phenotyping, reducing labor requirements
• Enable low-cost, quantitative phenotyping at scale across multi-environment variety trials, including direct in farmer fields
• AI-supported tools for multi-modal data collection and analysis enhance qualitative insights into farmer preferences and goals
• Inclusive innovation of breeding systems to produce high yielding varieties in target production environments, responsive to farmer preferences

## Graphical abstract

**Figure:**
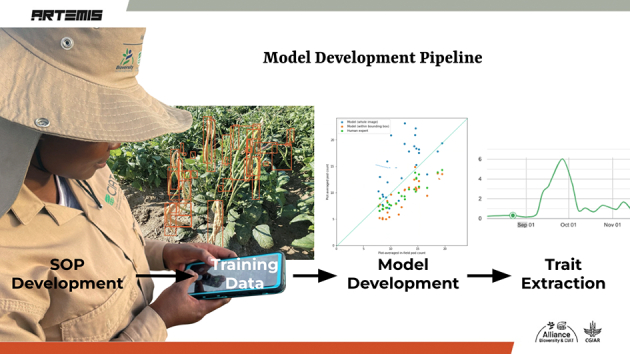

## 1 Introduction

The increase in genetic gain observed for improved crops in research settings often fails to materialize on-farm, particularly in low-resource smallholder systems across the Global South (Masuka et al., 2017). This gap arises in part from the logistical and biophysical complexities involved in evaluating germplasm performance in farmer production environments, coupled with the challenges of integrating farmer feedback into formal breeding processes. Adoption of improved varieties remains low in this context, suggesting, among other factors, a poor product-market fit between released germplasm and the production and consumption objectives of farmers and local markets (Ragot et al., 2018). In the Global South, where smallholder and subsistence farming remain the backbone of rural economies, rapid varietal turnover in farmer fields is a cornerstone of sustainable livelihoods and global food security (Atlin et al., 2017). Addressing this challenge requires transformational change in breeding systems. In a growing trend towards decentralization, public and private sector breeding programs are increasingly conducting trials in environments that better reflect the growing conditions of farmer fields (Ceccarelli and Grando, 2007). Participatory plant breeding (PPB) methodologies enhance these efforts by integrating farmer feedback at every stage of varietal development (Fadda et al., 2020). Yet large data requirements for accurate breeding remain a bottleneck for diversifying the conditions captured in breeding trials and eliciting nuanced and actionable feedback from farmers (Cobb et al., 2019). Technological developments in artificial intelligence (AI), deployed within an inclusive innovation framework, may be able to overcome major logistical barriers to on-farm breeding by facilitating high-throughput flow of information from farmers to breeders (Singh et al., 2016).

Computer vision (CV) technologies can replicate visual-estimation phenotyping via smartphone-deployable software – often exceeding the precision generated by classical methods (Xie and Yang, 2020a, Xie and Yang, 2020b). This technology has been around for at least a decade, but relegated to high-income breeding programs and requiring the use of expensive hardware (e.g. drones, robotics) and high-performance computational infrastructure (Peshlov et al., 2017). Recently, the release of large, open-source foundation models (e.g. DINOv2, LLaMA) has not only reduced the hardware requirements, but also lowered barriers to entry in terms of time and specialized skillsets needed to train and deploy models at scale. With this technology, high-quality quantitative phenotyping data can be collected by anyone with a smartphone, expanding the potential to measure crop performance in decentralized on-farm environments (Roitsch et al., 2019). Initial trials with common bean (*P. vulgaris*) support the validity of these tech-enabled approaches. Preliminary results suggest high correspondence of CV-based phenotyping with traditional analogue phenotyping methods for several traits, enabling performant direct-from-mobile imagery phenotyping models. Collecting unparalleled amounts of quantitative on-farm phenotype data could be key to unlocking mechanistic drivers of genotype by environment (GxE) interaction and enabling climate-resilient trait development in novel varieties for vulnerable farmers and environments. In parallel, novel AI-assisted data collection and analysis tools offer insights into the context and needs of farmers by eliciting farmer-prioritized crop traits to incorporate in breeder selection indices, leveraging farmers' expertise in identifying important visual indicators of crop performance and satisfaction. In this short communication, we explore pathways for transforming public sector breeding programs through the integration of AI tools for multimodal data collection and analytics. Targeting under-resourced programs in the Global South, these tools magnify the potential to produce locally adapted, high yielding and resilient seeds that meet the production and consumption objectives of resource-constrained farmers at decreased development time.

We situate our discussion within the framework of inclusive innovation for development (Heeks et al., 2013; Heeks et al., 2014; Swaans et al., 2014). In contrast to conventional models of innovation, which have focused on increasing the efficiency and productivity of high-income users, inclusive innovation seeks multidimensional impact for historically marginalized groups by engaging diverse stakeholders in a process of co-creation and reflexive learning (Heeks et al., 2014). If adopted, the technologies we discuss will transform the ability of breeding programs to effectively characterize seed users and target production environments, and may be the key to developing varieties that respond to the needs of previously excluded groups such as subsistence farmers, women, and those most vulnerable to climate change. In other words, the technologies will enable inclusive innovation of seed technologies by breeding programs, via the incorporation of robust farmer involvement in breeding decisions. However, achieving these outcomes also requires an initial phase of inclusive innovation within plant breeding institutions themselves. Resource-constrained breeding programs have historically been excluded from technological advances that have transformed plant breeding in other contexts. With unprecedented decreases in the cost and capital requirements of CV-based phenotyping and AI-assisted language processing, these technologies have become more accessible. Yet, integrating them into established institutional practices will require close collaboration with breeding teams in product design from the outset—directly considering the workflows, constraints, and incentives that govern plant breeding. We discuss the application of Human Centered Design (HCD) methodologies to achieve this, which involve ideation and iteration of proposed innovations with active involvement from target users, focusing on understanding their needs and capacities to identify appropriate solutions (Coggins et al., 2022; Holeman and Kane, 2020; Steinke et al., 2024).

## 2 Background

### 2.1 Inclusive innovations in crop breeding

Breeding data systems are complex. Globally, many breeding programs struggle to make effective use of basic analogue phenotyping data. Where resources are available, modern crop breeding in developed countries is a technology-intensive and data-driven process. Sophisticated sensors capture the details of observable plant characteristics, documenting the interaction of genetic variation with the environment, and breeders act on advanced computational insights to identify the most promising traits or combinations thereof (Araus and Cairns, 2014). In parallel, advances in market research align crop development with consumer trends, identifying desirable attributes that contribute to the uptake and commercial success of new varieties (Singh et al., 2006). However, these data-driven advancements in crop breeding haven't translated equitably across agricultural landscapes. Genetic gain and profits associated with innovations in data-driven crop breeding have largely accumulated in regions with technological advantages already in place, while agricultural yields continue to stagnate in the resource-constrained and marginal production environments that typify much of the Global South (Furbank and Tester, 2011). A major challenge at the core of this disparity is data availability, in part due to the logistical and biophysical complexities of measuring germplasm performance in farmer production environments and eliciting farmer feedback to inform selection. A centralized, top-down breeding paradigm does not cater to the reality of diverse and highly localized user profiles, as evidenced by the well-documented and systematic underperformance and under-adoption of improved seeds, particularly in smallholder systems (McEwan et al., 2021, Macours, 2019).

Public and private sector breeding programs have trended towards decentralization in recent years, pushing variety trials into environments that more closely resemble farmer fields and reflect the heterogeneity of agroecological conditions (Ceccarelli and Grando, 2007; Fadda et al., 2020; Singh et al., 2006). Decentralization efforts range from bolstering national breeding programs, where germplasm selected on local research stations is better adapted to the relevant soil and climate conditions compared to material developed internationally, to conducting variety trials directly in farmer fields. The more accurately breeding conditions approximate the target production environment of the end user, the more precisely the breeder can select for adaptive traits that provide localized agronomic advantage. The most challenging production environments, characterized by low input use, water stress, weed pressure, and degraded soils stand to gain the most from decentralized breeding, as they differ most substantially from agronomic research stations. Traits that may be adaptive to these conditions are unlikely to be selected for in on-station trials. While not intentional, many generations of parental breeding under ideal edaphic and other environmental conditions has inadvertently reduced genetic diversity of adaptations to real-world conditions, particularly those faced by smallholder farmers in the global south (Renzi et al., 2022).

Concurrently, increasing emphasis is placed on diversifying the user profiles considered for variety development by soliciting qualitative feedback directly from farmers who interact with novel germplasm through participation in crop trials. Together, these participatory plant breeding (PPB) methodologies integrate the efficiencies of modern breeding programs in accelerating genetic gain through genomics and phenotyping technologies with decentralized farmer involvement. This approach serves to identify local preferences and climate-adaptive traits, diversify genetic material with farmer varieties and landraces, and evaluate improved varieties in-situ (Fadda et al., 2020). By incorporating stakeholder input at every stage of the crop improvement process, PPB can deliver highly targeted variety recommendations built around farmer preferences, and promote adoption by engaging farmers in the selection and evaluation of high-quality seeds uniquely suited to their on-farm needs.

These innovations can be described in terms of the ladder of inclusive innovation, a framework put forth by Heeks et al. (2014) to define the levels of inclusivity, “with each succeeding step representing a greater notion of inclusivity in relation to innovation.” First, an innovation can be inclusive with respect to its intention, i.e. to address the needs of an excluded or marginalized group. The second level, consumption, is reached if the innovation is in fact adopted by the intended users, and third level, impact, requires that the innovation has positive outcomes for adopters. Seed technologies developed through decentralized plant breeding are intended to serve farmers whose needs have not been met by conventional varieties bred to maximize yields under optimal production conditions. Early evidence suggests that adoption of the resulting varieties is high, and impact has been measured along several socioeconomic dimensions (Fadda et al., 2020; Teeken and Temudo, 2021; van Etten et al., 2023). The fourth level of inclusivity pertains to the process: “an innovation is inclusive if the excluded group is involved in the development of the innovation” (Heeks et al., 2014). This gets at the core of decentralized plant breeding - running farmer-managed variety trials in diverse on-farm environments, and incorporating farmer feedback to identify priority traits for distinct user groups. Levels five and six regard the inclusiveness of the institution leading the innovation and the frame of knowledge and discourse surrounding it. The CGIAR research centers who are leading public sector innovation in this domain have a mandate to “Improve the food system to ensure an adequate and nutritious diet, especially for the world's most vulnerable people.^1^”

### 2.2 The phenotyping bottleneck

Despite strong theoretical advantages and promising results from early implementations, quantity and quality of plant performance data remains a critical bottleneck to decentralized PPB at scale, particularly for resource-constrained breeding programs in the Global South. Historically, logistical limitations of phenotyping relegated most selection decisions to the research station, where crop trials require intensive phenotypic data collection. In low-resource breeder settings, phenotyping is carried out manually by trained technicians who are often in short supply and high demand by national breeding programs. Technicians count pods, measure leaves, and score disease using a combination of rigorous techniques and subjective intuition. The data is collected at a high cadence and analyzed by the breeder to inform which parents will be used for future crosses. This process has substantial room for error and inconsistencies across individuals. Considering these major challenges to data collection even on the research station, quantitative analogue phenotyping across decentralized multi-environment trials (METs) is a non-starter.

As trials move farther from research hubs and multiply across locations, resources are stretched thin, necessitating tradeoffs. Successful MET methodologies often sacrifice depth of data for breadth. For example, triadic comparisons of technology, or tricot, is a scalable approach to on-farm trials where participating farmers grow and evaluate three varieties on their own farm, using an incomplete block design wherein many more varieties can be compared overall (Van Etten et al., 2019; Van Etten et al., 2020; De Sousa et al., 2021a; de Sousa et al., 2021b; Steinke and van Etten, 2016). Farmers qualitatively rank each variety along various attributes, providing guidance for the release and marketing of varieties to appropriate target environments based on farmer preferences. While tricot works well for qualitative evaluation of non-segregating pre-release germplasm, it does not provide the quantitative phenotyping data required at earlier stages of breeding, necessary to make informed crossing and selection decisions. Acquiring this type of data using analogue methods would entail sending trained technicians on a weekly or even daily basis to hundreds or thousands of trial sites spread across the target population of environments. The largest proportion of trait segregation and selection pressure occurs during early phases of the breeding process, yet it is only at the last stages of the varietal development process where some breeding programs integrate on-farm evaluative feedback (Cobb et al., 2019). At these later stages, most of the genetic characteristics governing plant performance are fixed. Many programs omit this step altogether if the attainable qualitative data alone does not warrant the cost of an on-farm trial.

### 2.3 Technology-supported quantitative phenotyping: Progress and limitations

The limitations of analog phenotyping on-station have been effectively overcome by technology in state-of-the-art breeding settings. Digital phenotyping enables unprecedented levels of speed, accuracy, and objectivity in crop assessment (Furbank and Tester, 2011). Beyond improving the quality and quantity of phenotypic data, digital phenotyping opens the door to types of data that physically could not be collected by hand, for example continuous time series of the flower count across an entire field over the growing season. As selection accuracy is one of the main drivers of genetic gain in breeding programs, reduced error via CV has strong implications for genetic gain and shortening of breeding cycles. Higher selection accuracy can better inform breeding decisions to eliminate a larger portion of crosses and progeny earlier based on subtle but meaningful insights derived from higher quality phenotypic data (Cobb et al., 2019).

However, no amount of phenotypic data from a static or controlled setting can replace the insights gained from observing crops in their target environments. There is a high level of environmentally-mediated phenotypic plasticity in many crops that is not observable in highly managed trial station locations (Brooker et al., 2022). The same variety that performs well in breeder settings with abundant nitrogen and irrigation may underperform in drought conditions or nutrient-poor soils often found in smallholder systems, relative to another variety that is bred for resilience over potential yield. Indeed, the genetic gains measured in research stations are substantially lower when evaluated on-farm, confirming the pivotal role of environmental interactions in shaping crop performance outcomes (Magorohosho et al., 2010). While some environmental pressures can be replicated or simulated for a trial, capturing the influence of the local environment on variety performance can only occur in-situ due to the complexities of real-world environments and cropping systems.

High caliber quantitative data from farmer field trials will shed light on the complex interactions between genotype, environment, and management (GxExM), and guide breeders to optimize germplasm for diverse production conditions (de Sousa et al., 2021b). Advanced CV phenotyping technologies will supercharge decentralized breeding methodologies and the production of seeds tailored for varied and extreme production environments. This supports marginalized groups today and prepares seed systems for the changing climates of the future. A corps of research at the Alliance aims to democratize advanced phenotyping technologies and other AI-powered tools for multimodal data collection by extending access to anyone with a smartphone, opening the door to transformative data-driven insights into crop-environment interaction. The ladder of inclusive innovation (Heeks et al., 2014) is a useful framework here as well, with resource constrained breeding programs in the Global South as the group historically excluded from technological advances. Appropriate tools that leverage the state of the art AI technologies for plant breeding in this context must be developed by and for the intended users through an inclusive, human-centered design process to ensure adoption and impact (Table 1).

**Table 1:** Plant stand count CV model accuracies (Pearson Correlation) relative to in-field ground-truth across multiple evaluation locations. Multiple Plant are single images that contain between three and four plants. Whole Plot are single images that encompass the entire breeding plot (4 m × 5 m) into one image. Panorama are a composite mosaic of multiple images taken across one plot.

	Multiple Plant	Whole Plot	Panorama
Colorado	0.84	0.96	NA
Tanzania	0.99	0.85	0.96
Colombia	0.85	NA	0.72

## 3 Materials and methods

### 3.1 Mobile phenotyping tools

Housed within the digital inclusion arm of the Alliance, the Artemis project is developing mobile phone-based imagery phenotyping tools to support CGIAR and associated National Agriculture Research Systems (NARS) breeding programs in the Global South. A core innovation for enabling high-throughput digital phenotyping both on- and off-station, mobile phenotyping tools have transformative potential to enhance and expand the datasets that inform crop improvement processes. These tools can augment and replace analogue phenotyping on research stations, enhancing the accuracy, objectivity, and consistency of measurements while reducing labor requirements. We have measured imagery-modeled trait accuracies with Pearson Correlation values between 0.88 and 0.95 with ground truth data, with the ability to fully phenotype a breeder's plot (4 m × 3 m) in under a minute. Moreover, they open the door to unprecedented expansion of data collection to remote and heterogeneous agricultural settings.

#### 3.1.1 Leveraging computer vision

Success for breeding programs supported by mobile imagery-based phenotyping involves increased selection accuracy powered by CV trait imagery models. Initially, imagery models learn from large quantities of images sourced from rovers and mobile-image trials. The data is manually labeled by agronomic experts and data technicians, which implies substantial upfront costs for adapting models to new crops or environments. However, recent advancements in AI offer ways to mitigate the cost of operationalized image-based phenotyping by making it possible to train trait imagery models with significantly fewer images than before, and reducing labeling requirements. Specifically, by employing state-of-the-art foundation models such as DdinoV2 (Oquab et al., 2023) and Segment Anything (Kirillov et al., 2023), trait imagery models can now be trained on just a few hundred images. Foundation models are pre-trained on vast datasets, allowing them to recognize intricate patterns and generalize knowledge across domains. Few-shot learning techniques enable models to learn from a small amount of labeled data, while zero-shot learning allows them to infer patterns and make predictions for unseen or novel classes without any labeled examples. Ultimately, these advances will enable breeding program staff with limited technical expertise to swiftly and effectively deploy flexible phenotyping models tailored for specific crop-trait applications, for example, parasitic weed detection on sorghum in West Africa.

#### 3.1.2 Refining image collection SOPs

Model performance still remains subject to image quality, which may be challenging to standardize on-farm or globally across multiple breeding stations. Successful image recognition requires clear and consistent images of plants throughout the growth cycle. While existing models work well with standardized, high-quality images taken from crops in a regulated growth environment, collecting operational images from field sites is a non-trivial challenge. Complications inherent to low-resource research settings, such as the presence of multiple crop diseases and nutrient deficiencies, inconsistent field layouts, and lower quality hardware for image collection can compromise model performance. In the highly decentralized multi-environment trials, where the impact of mobile phenotyping data may be strongest, there is the additional challenge of hiring and training locally-based staff to collect images. Breeder trials have traditionally been spatially constrained by the substantial costs of sending trained technicians to environments far from the research base. The introduction of mobile phenotyping drastically reduces the requirements of specialized skillsets for data collectors. An ongoing pilot study uses a decentralized enumerator model, in which recent college graduates are employed to manage data collection from clusters of on-farm trials in their own villages. In this initial round, enumerators were trained in-person at a central location, but we are developing digital instruction materials to enable fully remote training, eliminating all but local transportation costs. However, clear protocols are required to train individuals to collect images of sufficient quality to meet model requirements with minimal oversight. All of these challenges can be met through a dual approach: defining straightforward, intuitive standard operating procedures (SOPs) for image collection (which may be guided by tailored user interface software), and fine-tuning machine learning models using more diverse, field-realistic training datasets.

SOP development requires a tight feedback loop between breeders, field technicians, enumerators, and AI engineers. Breeding traits are highly specific, for example, disease scoring of lower leaves in early season crops, and flower count during early reproductive stages. Imagery types need to replicate what field technicians ‘see’ in the field: angle of view, time of day, etc. AI engineers use these images to determine how image-type affects model quality with respect to the trait of interest for the breeding programs. For example, image angle, soil color, time if day the imagery were collected, etc. Once the optimal image type has been identified, field technicians are required to determine the best method for effective execution (considering both time and tedium). Appropriate methods may differ on and off station, for example on-station phenotyping may prioritize data quality and quantity per trial, using more capital intensive and complex methods such as push-carts, panorama images, and specialized angles. If the goal of on-farm phenotyping is to get basic quantitative data from as many environments as possible, simpler SOPs that can be implemented with minimal training may suffice. This entire process needs to be replicated per crop species and trait, and is only successful if the interactions between all users are open and fluid. An inclusive innovation approach integrates breeders, technicians, and enumerators from the earliest stages in development and delivers an end product built through a process of co-creation, giving all a sense of ownership and laying the groundwork for meaningful adoption and impact within breeding programs.

#### 3.1.3 Integrating Mobile phenotyping with existing breeder workflows

Much like the consideration given to farmers in the inclusive development of seeds and crop varieties, the intended users of a new digital toolkit need to be integrated from the onset. Many technologically advanced interventions falter when the goals and capacities of the existing system are not adequately assessed, frequently imposing solutions to problems that may not be perceived as pressing by users (Heeks et al., 2014; Steinke et al., 2022; Masiero, 2016; Krell et al., 2021). Identifying specific user needs can make digital tools more relevant and likely to be adopted. Limited digital literacy and skillsets present a further challenge to the introduction of technologies if users are reluctant to take them on (Agyekumhene et al., 2020; Radovanović et al., 2020). New tools should be user-friendly, catering to users' demands and acknowledging varying digital capacities and infrastructure. Prioritizing simplicity over flexibility is key, and sticking as closely as possible to existing manual workflows can reduce user burden and avoid unnecessary complexities (McCampbell et al., 2021; Steinke et al., 2022; Antonini, 2021). Phenotyping is already a laborious process, and a new tool is unlikely to be adopted if it increases workload or does not improve outcomes in a way that is apparent and relevant to users. These considerations are imperative for the proposed innovations to reach the second and third levels in the ladder of inclusivity, passing from intention to adoption by breeding programs, and eventually impact.

**Fig. 1:**
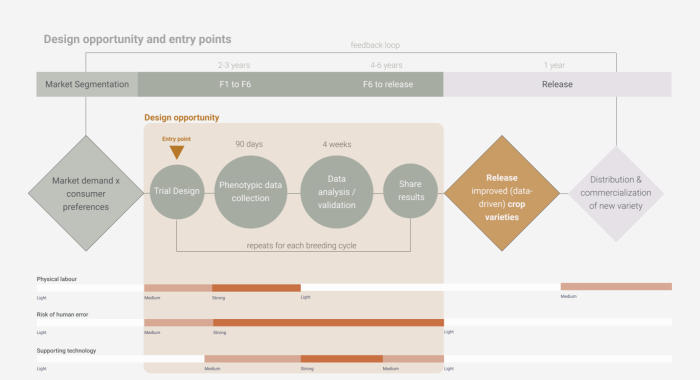
Entry points for a digital solution throughout the breeding cycle.

Following a HCD approach to technology development, a series of interviews and observations were conducted with breeders, breeding technicians, on-farm trial enumerators, and other support staff in Tanzania to understand the breeding ecosystem and identify motivations and pain points throughout the process. Archetypal ‘user profiles’ were distilled from these interviews to better characterize users' needs and capacities, and to identify entry points for the integration of imagery phenotyping tools to improve and facilitate existing workflows. A detailed ‘breeder's journey’ map was developed to elucidate specific tasks that can gain efficiency from digitalization. These tasks require high physical labor, are prone to human error, and lack supporting tools and software, including: trial design, phenotyping data collection, data analysis, and results sharing (Fig. 1). This process revealed that a viable mobile phenotyping tool should meet the breeder-demand of increased data accuracy, reduce labor requirements for technicians, and contain clear instructions for users with limited digital literacy.

We are currently exploring ways to leverage multimodal datasets to further efficiently execute standardized SOPs on-farm and ease the transition to digital phenotyping for breeding staff. Recent advances in Large Language Models (LLM) such as GPT4 from Open AI and LLaMA from Meta have been able to replicate more interactive and conversational approaches to interacting with technology. By integrating CV with Natural Language Processing (NLP) and LLMs we may be able to provide a more intuitive way to support SOP execution and data quality. In this scenario, the LLM will instruct users of the mobile phenotyping tool to collect images using a specific SOP. The CV model will analyze the data quality and if needed for enhancement will request the user to collect additional images. This process can be repeated until data of sufficient quantity and quality are collected. Integration with NLP can close the loop by allowing the user to input audio data corresponding to issues present in the field (e.g. pests or diseases). These attributes further reduce the barriers to entry and specialized skillsets necessary to collect and analyze high quality data, and can be used in conjunction with digital training materials to on-board phenotypers and moderate data collection across locations with little to no in-person oversight.

## 4 Results and discussion

### 4.1 Preliminary results from mobile phenotyping trials

#### 4.1.1 Optimization for accuracy

**Fig. 2:**
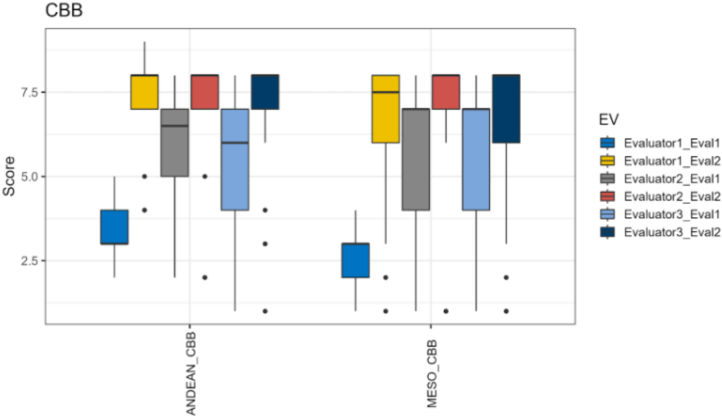
Visual-based disease scores for common bean bacterial blight among Alliance pathologists. Score is a 1–9 scale. Two different bean populations were tested (Andean and mesoamerican). Three different evaluators were considered (evaluator 1 was in training and evaluators 2 and 3 were experts) and two timing points (Eval1 and Eval2 for each evaluator).

It is widely acknowledged that there are a range of inconsistencies common to manual visual phenotyping (Cobb et al., 2013; Furbank and Tester, 2011). The process is highly subjective, and the accuracy is influenced by multiple factors including the experience of the evaluator, the time of day affecting fatigue levels (cool mornings vs. hot and sunny afternoons), and overall tedium associated with phenotyping hundreds to thousands of plots in some breeding programs. Though recognized, these inconsistencies are rarely quantified. We have analyzed phenotypic disease scoring data for common bean taken by highly trained technicians and pathologists in the Alliance fields in Cali, Colombia, and found significant variation in scores by these professionals (Fig. 2). In contrast, CV-based estimates from mobile-phone crop images obtained high-levels of accuracy relative to absolute ground truth (i.e. measuring every plant in every plot by hand). In the specific case of disease, there is no absolute ground truth, but the CV models can estimate leaf area and segment the percent infected pixels per leaf, something human eyes are incapable of achieving.

**Fig. 3:**
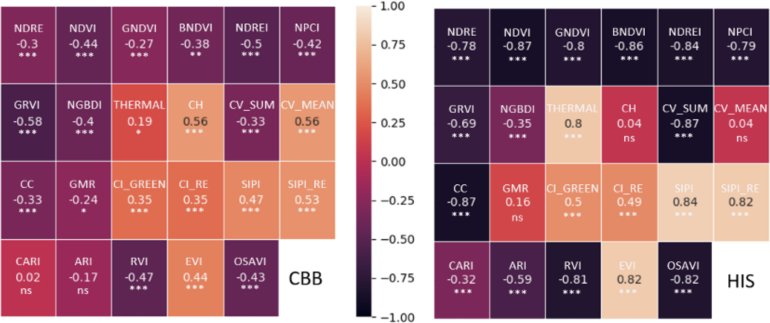
Comparison of visual disease score estimations (CBB) and plant health index measurement (HIS) using drone imagery. The left side of the figure (CBB) and the right side (HIS) illustrate the correlation between the ground-truth visual score and the drone-derived metrics. The metrics derived from RGB images include Canopy Cover (CC), Canopy Volume (CV), and Canopy Height (CH), while other Vegetation indices are derived from multispectral images.

One of the benefits of imagery-based phenotyping is consistency in measurement. Provided clear SOPs and imagery quality, machines produce modeled trait outputs of consistent quality. Further, models can integrate multiple computer vision estimates that are highly correlated with the best in-field visual assessment. For example, we have validated that a CV-based plant health index (drone executed) can equal the accuracy of the best human disease score estimates (Fig. 3). One of the many benefits is that these imagery tasks can be executed throughout the day or at a high cadence throughout the growing season to provide near continuous data generation.

#### 4.1.2 Throughput efficiency and reduced labor

**Fig. 4:**
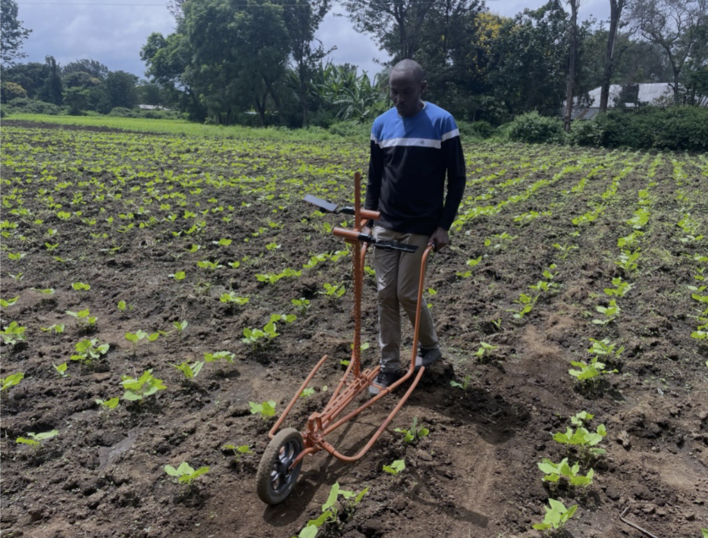
Companion phenotyping device (Tanzania).

For mobile phone-based imagery phenotyping, accurate models are necessary, but not sufficient for full integration in breeding programs. Considerations have to be made for throughput efficiency in addition to accuracy. In the Artemis project, initial manual SOPs required 12 person-hours to fully phenotype 30 breeding plots. This is an order of magnitude more than traditional visual estimations. Labor is the limiting factor in this scenario. To overcome this challenge, technicians worked again with AI engineers to test and develop low-cost and low-tech companion devices (Fig. 4) to aid the efficiency of phenotyping throughput. With this device, technicians are able to phenotype an entire breeding plot (4 m × 3 m) in less than 30 s, exceeding manual visual methods.

#### 4.1.3 Increased data quantity on-station and on-farm

The increased efficiency of digital phenotyping relative to analogue methods vastly increases the amount of data that can be collected, both within and across trials. Within a given trial, digital phenotyping enables technicians to capture phenotypic data from every plant (analogue methods typically involve random selection of plants to measure), and at much higher cadence. On initial on-station trials on common bean at the Alliance office in Tanzania, plots are imaged daily. This has increased the quantity of data from around a dozen measurements per genotype per season with traditional visual estimation by about one order of magnitude with imagery-based methods. As discussed, a key advantage of digital phenotyping is the ability to collect data from any trial using only a smartphone. The ongoing on-farm pilot study described in Section 3.1.2 has successfully captured images and ground truth for two traits - stand count and pod count - from 480 trial sites across five districts in Tanzania, spanning distinct agro-ecologies. To our knowledge, this is the first digital phenotyping data ever to be collected from an on-farm trial, and the first quantitative phenotyping data in general to be collected from such a large scale and spatially diverse on-farm trial.

### 4.2 Mechanisms for farmer feedback

Beyond improving yields in farmer production environments, innovations in digital data collection can bolster seed system inclusivity by strengthening communication between farmers and formal breeding programs. Historically, elicitation of farmer feedback required costly and time-consuming structured surveys. While informative, this format is limited by logistical constraints on the depth and breadth of information that can be collected. Leveraging innovations in LLMs to integrate automatic speech recognition and chat functionality into on-farm trials may better capture farmers' opinions in natural language, potentially providing greater insight than text-based approaches (Hase and Nimbhore, 2023; Becker, 2016; Jones-Garcia, 2022). As smartphone penetration and digital literacy increase, this technology could even bypass the need for an in-person survey, with farmers interacting directly with a mobile survey tool. With these techniques, we can collect valuable and previously inaccessible insights into the preferences, priorities, and challenges faced by farmers, which can inform current and future crop breeding strategies and ultimately improve livelihoods. This approach may provide farmer feedback on the traits collected during the phenotyping process, prioritizing traits that matter most to farmers which can be considered in breeder selection indices.

**Fig. 5:**
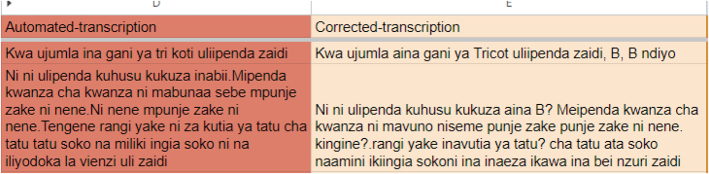
Automated transcription using finetuned Whisper model.

NDIZI (NLP to Develop and Innovate Zero-Shot Intelligence), a sister project to Artemis, is leveraging the same on-farm pilot study introduced in Section 3.1.2 to explore the feasibility and effectiveness of NLP and LLMs in collecting farmer feedback from field trials across different agro-ecological zones in Tanzania, enabling real-world testing and validation. Interviews have been conducted with smallholder farmers to collect audio feedback from trial participants, testing different methods for prompting and facilitating meaningful dialogue around variety preferences The recorded audio, predominantly in Swahili, is then transcribed to create the necessary training data for fine tuning and evaluating automatic speech recognition models (ASR) and NLP models for analysis and extraction of traits. In particular, we finetune Whisper, a multilingual foundation model for ASR trained on 680,000 hours of multilingual and multitask supervised data collected from the web. While the initial results are promising, additional data is required to further improve the model performance, as shown in Fig. 5. The final NLP model will identify traits mentioned by farmers (trait extraction), produce a score for each variety with respect to each trait mentioned - e.g. yield scored from 1 to 5, and distinguish between a range of positive to negative attitudes towards each trait (sentiment analysis). The model will also produce a ranking of traits in order of importance for each farmer, as well as a proxy ranking for the entire sample or subsets (e.g. women, drought-prone, etc) based on the number of times a trait was mentioned. These methods reproduce and expand on the gold-standard in participatory variety selection (see e.g. Kolech et al., 2017; Nchanji et al., 2021; Van Etten et al., 2019) increasing both the quality (depth) and quantity (breadth) of attainable farmer feedback.

In combination with speech data, we are also collecting image data from which we learn about the visual indicators of crop performance that are noticed by farmers, thereby integrating farmers' knowledge of crop selection to improve the phenotyping technology (Ragot et al., 2018). This multimodal data provides insights on how farmers relate to their bean crops and evaluate new varieties. An explicit research focus is placed on the manner in which farmers articulate their needs, including communication style (e.g. questions vs storytelling), wording, socioeconomic markers of speech such as gender and age, and non-verbal cues such as facial expressions and tone of voice which may influence the meaning of what is being expressed. By identifying nuanced response patterns, the research aims to refine digital data collection methods and extend the scope of in-depth qualitative processes to a broader range of participants. Besides classical machine learning, prompting of large foundation models, with multimodality capabilities, is applied to perform trait extraction and assist the flow of dialogue between farmers and enumerators, suggesting flexible follow-up questions based on response patterns beyond the pre-set skip-logic available in hardcoded electronic questionnaires.

## 5 Conclusion

The emergence of AI tools for high-throughput multi-modal data collection and analysis offers a unique opportunity for equitable and inclusive transformation of crop breeding systems. Inclusive innovation, which involves the development of goods and services both for and by historically underserved communities, is at the core of the envisioned transformation (Heeks et al., 2014; Opola et al., 2021). Moving towards technology-enabled participatory breeding systems, farmers are actively integrated into the variety improvement process from the earliest stages and play a key role in driving the development of genotypes that are adapted to their specific biophysical and socioeconomic conditions. Prioritizing stakeholder engagement in digital knowledge creation empowers breeding systems to respond in a data-driven way to localized cultural preferences, food security requirements, and agroecological conditions (Birhane et al., 2022). The technology is ready to facilitate this fundamental shift, but must be met with a shift in traditional mindsets about how breeding is done. The inclusive innovation model for technology development can ease this transition, ensuring that new tools are easy and appealing to use and address a relevant challenge. Engaging the broader plant breeding community is crucial for large-scale adoption of new methods, and for concurrent advances in seed accessibility and information transfer required for meaningful systemic change.

While the transformative potential of these technologies exists within the domain of inclusive crop improvement, to our knowledge no technology solution has been successfully deployed at scale. While digital capacities across the Global South are increasing, substantial barriers to technology access remain for much of the rural population, including high costs for mobile phone handsets and data, lack of relevant content in local languages and dialects, and low digital literacy (Digital Frontiers, 2023). However, the technology landscape is not static, the tech startup ecosystem is growing across most of the Global South with indications that developing countries may become the largest growing market for technology (Lambrechts et al., 2021). Inclusive innovation and human centered design will be of greater importance given this dynamic nature of technology development (Foster and Heeks, 2013).

Development and maintenance costs to set up an AI-enabled phenotyping system are additional unknowns. Engineering time and cloud-compute infrastructure to build and maintain a system operating at the global-level will be substantial. A rough estimate is on the order of a few million USD per year. While the costs and challenges are high, these technologies may be transformative towards the rapid development of locally-adapted and climate resilient crop varieties for vulnerable populations. The costs of inaction may be many orders of magnitude more than the fixed and variable costs to build and maintain the phenotyping system. Finally, considerations have to be made regarding the social and ethical impacts of using AI-enabled tools with smallholder farmers (Ryan, 2022). Many of the large and open-source foundational models were not trained on data generated by the target beneficiaries of the work presented here. There is concern that ‘out-of-the-box’ usage of these general models may provide a bias along gender, ethnicity, or socio-economic dimensions. As developers of AI for agriculture, it is important to ensure that HCD and inclusive design approaches account for these considerations. As this field of technology is rapidly evolving it is more important than ever to address social and ethical issues during the development process.

## CRediT authorship contribution statement

**Violet Lasdun:** Conceptualization, Data curation, Formal analysis, Investigation, Writing – original draft. **Davíd Güereña:** Conceptualization, Funding acquisition, Project administration, Supervision, Writing – review & editing. **Berta Ortiz-Crespo:** Investigation, Writing – review & editing. **Stephen Mutuvi:** Data curation, Formal analysis, Funding acquisition, Investigation, Methodology, Validation, Writing – review & editing. **Michael Selvaraj:** Data curation, Formal analysis, Investigation, Methodology, Validation. **Teshale Assefa:** Investigation, Methodology.

## Declaration of generative AI and AI-assisted technologies in the writing process

During the preparation of this work the authors used ChatGPT(gpt-4) in order to format references. After using this tool/service, the authors reviewed and edited the content as needed and takes full responsibility for the content of the publication.

## Declaration of competing interest

We, the authors, declare that we have no conflict of interest, financial or otherwise, in the content, views, conclusions, or opinions of this manuscript.

## Data Availability

Data will be made available on request.

## Footnotes

1 https://www.cgiar.org/introduction/
